# Multiple Comparison of Age Groups in Bone Mineral Density under Heteroscedasticity

**DOI:** 10.1155/2015/426847

**Published:** 2015-08-27

**Authors:** Ahmet Sezer, Lale Altan, Özer Özdemir

**Affiliations:** ^1^Department of Statistics, Faculty of Science, Anadolu University, 26470 Eskisehir, Turkey; ^2^Department of Physical Medicine and Rehabilitation, Medical Faculty, University of Uludag, 16059 Bursa, Turkey

## Abstract

Osteoporosis is a silent disease because individuals may not know that they have osteoporosis until their bones become so fragile. Bone mineral density (BMD) test helps to detect osteoporosis and determine the risk fractures. This study covers bone measurement data from total body dual energy X-ray absorptiometry scans for 28,454 persons who participated in the 1996–2006 National Health and Nutrition Examination Survey in USA Dual energy X-ray absorptiometry (DXA) method is known as the primary method for detecting osteoporosis because of its high precision and accuracy. Testing the equality of the means of normal populations when the variances are unknown and unequal is a fundamental problem in clinical trials and biomedical research. In this study we compare age groups based upon BMD in case of unequal variance being present among the groups. First we test equality of variances among the age groups by the Hartley test. And then Scott-Smith test is used to test equality of BMD means for the age groups. Finally, Tukey-Cramer confidence intervals are constructed to detect which groups start to differ from the reference group in which BMD reaches the peak level.

## 1. Introduction

Osteoporosis is a systemic skeletal disease characterized by low bone density and micro architectural deterioration of bone tissue with a consequent increase in bone fragility [1]. Measurement of BMD can be used to determine fracture risk and monitor the effects of treatment. Early detection of bone loss is essential to preventing osteoporosis. In fact, osteoporosis affects more than 75 million people in Europe, Japan, and USA and causes more than 2.3 million fractures annually in Europe and USA. The lifetime risk for hip, vertebral, and forearm (wrist) fractures has been estimated to be around 40%, very close to that for coronary heart disease. Osteoporosis does not only cause fractures, but also causes people to become bedridden and causes back pain and loss of height. Prevention of the disease and its associated fractures is important for maintaining health, quality of life, and independence among the elderly.

Early osteoporosis is not usually detected and most of the time does not become clinically evident until fractures occur. Loss of bone density occurs with advancing age and rates of fracture increase with age, giving rise to significant morbidity and mortality. Osteoporosis is three times more common in women than in men, because women have a lower peak bone mass and hormonal changes occur at the menopause. Estrogens have an important function in preserving bone mass during adulthood, and bone loss occurs as levels decline, usually around the age of 50 years. In addition, women live longer than men [2] and therefore have greater reductions in bone mass.

Until recently, osteoporosis was considered an inevitable consequence of ageing. With Improvements in diagnostic technology and assessment facilities, now it is easier to detect the disease before fractures occur. The substantial bone loss is usually around age 65 years in men and 50 years in women [3]. Females tend to keep peak mineral content until menopause; after that it drops about 15% per decade. Fracture rates increase rapidly with age and the lifetime. Many studies show that an inadequate supply of calcium over a lifetime contributes to the development of osteoporosis. The body's demand for calcium is greater during childhood and adolescence and during pregnancy and breastfeeding. Epidemiological studies indicate that a 10% increase in peak bone mass in the Caucasian female population would decrease the risk of hip fracture by about 30%. Clearly, eliminating the risk factors might significantly reduce the burden of osteoporosis. Obvious interventions include raising levels of exercise, stopping smoking, and increasing dietary intake of calcium [4].

## 2. Materials and Methods

Testing the equality of the means of normal populations when the variances are unknown and unequal is a fundamental problem in clinical trials and biomedical research. A well known case is the Behrens-Fisher (BF) problem, which focuses on two populations. Behrens-Fisher problem is popular because there is no exact solution satisfying the classical criteria for good tests. The problem is seemingly simple, yet much effort has been made to try to solve this problem [5–7].

It is well known that there exists an analysis of variance (ANOVA) *F*-test for the problem of testing the equality of means from several independent samples under the assumptions of normality. It is well known that the usual *F*-test is not robust to the assumption of equal variances. There is, however, no standard procedure for testing this hypothesis when variances are not equal, and various approximate test procedures have been proposed in the literature. The best known procedure is the test proposed by Welch (1947) [8] and its modifications. Other tests have been proposed by James (1951) [9], Scott and Smith (1971) [10], Brown and Forsythe (1974) [11], S.-Y. Chen and H. J. Chen (1998) [12], Rice and Gaines (1989) [13], Krishnamoorthy et al. (2007) [14], Weerahandi (1995) [15], and Xu and Wang (2008) [16, 17].

### 2.1. Hartley's Test for Testing Variance

Hartley's test (*F*) was developed by Hartley in 1950 [18]. This test assumes that data within each group are normally distributed and test involves computing the ratio of the largest group variance (max⁡*s*_*i*_^2^) to the smallest (min⁡*s*_*i*_^2^). This ratio will be compared with the critical value from a table of the sampling distribution of *F*. In this test null hypothesis states that all groups have equal variances alternative to at least one group differing from the others. Consider

(1) $$\begin{array}{c} F=\frac{\max s_{i}^{2}}{\min s_{i}^{2}}\;i=1,\ldots ,k, \end{array}$$

where *k* is the number of the group.

Since we have large sample sizes for the groups, we used critical value 1 from Hartley's table to make our decision. For example Hartley test is calculated for the first group, Non-Hispanic Mexican-American female group; consider

(2) $$\begin{array}{c} F=\frac{{\left(0.125\right)}^{2}}{{\left(0.084\right)}^{2}}=2.21>1. \end{array}$$

Since calculated test statistic value is bigger than 1 (critical value from the Hartley table), we reject the null hypothesis that equality of variance assumption is violated among age groups of Mexican-American females. It is evident that equal variance assumption is violated for all the cases we consider (Tables 1 [Table tab2] [Table tab3] [Table tab4] [Table tab5]–6) and that leads us to check equality of means under heteroscedasticity.

### 2.2. Tests for the Means

A very common problem in applied statistics is that of comparing the means of several populations. Several methods have been proposed for handling the unequal variance among the groups: Welch test, Brown-Forsythe test, and Scott-Smith test. Since we have two classes for gender and three classes for the race, totally there are 6 tables. Each table includes sample size, sample mean, and standard deviation for the groups. In the null hypothesis we claim all age groups have the same mean of BMD. On the other hand alternative claims at least one group has different mean than the rest of the groups. Consider

(3) H0: μ1μ2=⋯=μk=μ,H1: ∃μiμj,1≤i≤j≤k.

#### 2.2.1. The Scott-Smith Test

In this study, because of its simplicity Scott-Smith test will be used for checking equality of BMD means for the age groups. Scott and Smith (1971) give the following test statistics to test the means under heteroscedasticity [10]. Consider

(4) $$\begin{array}{c} F_{s}=\overset{k}{\underset{i=1}{\sum }}\frac{n_{i}{\left({\overset{-}{X}}_{i}-\overset{-}{X}\right)}^{2}}{S_{i}^{*2}}, \end{array}$$

where *S*_*i*_^*∗*2^ = (*n*_*i*_ − 1)/(*n*_*i*_ − 3)*S*_*i*_^2^. Under the null hypothesis distribution of *F*_*s*_ will be *χ* ^2^ with degrees of freedom *k*.

#### 2.2.2. The Welch Test

Welch (1951) [19] generalized the test which is proposed to handle Behrens-Fisher problem. Because of its simplicity Welch test is commonly used in practice. According to Welch, the test statistics is

(5) W=∑i=1kwiX−i−X−2/k−11+2k−2/k2−1∑i=1k1/ni−11−wi/∑wj2,

where *w*_*i*_ = *n*_*i*_/*S*_*i*_^2^. Under the null hypothesis, *W* statistic has *F* distribution with *k* − 1 and *f* degrees of freedom, where

(6) $$\begin{array}{c} f=\frac{1}{\left(3/\left(k^{2}-1\right)\right)\sum_{i=1}^{k}\left(1/\left(n_{i}-1\right)\right){\left(1-w_{i}/\sum w_{j}\right)}^{2}}. \end{array}$$

We reject the null hypothesis when *P*(*F*_*k*−1,*f*_ > *w*) < *α*, where *w* is calculated test statistic from (5).

#### 2.2.3. The Brown-Forsythe Test

Brown and Forsythe (1974) [11] modified classical *F* test. The proposed test statistic is

(7) $$\begin{array}{c} B=\frac{\sum_{i=1}^{k}n_{i}{\left({\overset{-}{X}}_{i}-\overset{-}{X}\right)}^{2}}{\sum_{i=1}^{k}\left(1-n_{i}/n\right)S_{i}^{2}}. \end{array}$$

Under the null hypothesis this test statistic has *F* distribution with degrees of freedom *k* − 1 and *ν*, where *ν* is

(8) $$\begin{array}{c} v=\frac{{\left[\sum_{i=1}^{k}\left(1-n_{i}/n\right)S_{i}^{2}\right]}^{2}}{\sum_{i=1}^{k}\left({\left(1-n_{i}/n\right)}^{2}S_{i}^{4}/\left(n_{i}-1\right)\right)}. \end{array}$$

Brown-Forsythe test rejects the equality of mean hypothesis when *P*(*F*_*k*−1,*v*_ > *b*) < *α* where *b* is the calculated test statistic from (7).

Finally, the Tukey-Cramer confidence intervals are constructed to determine which age groups are statistically different than the reference group. The reference group will have the highest BMD mean among the groups. If Tukey-Cramer pairwise confidence interval does contain zero, it means that there is no statistically significant difference between the considered groups with respect to their BMD. The first age group in the last column of the tables will represent the highest group with BMD mean (for females: X5, for males: X4). For example, in Table 1, the Mexican-American female group, 30–39-year (X5) group, has the highest mean with respect to BMD. Since Mexican-American females reach their BMD in 30–39-year (X5) group, this group will be our reference group and we will compare other age groups with this reference group.

## 3. Results and Discussion

Our study reveals that BMD decreases rapidly with age for both sexes and in all race groups. After 50 years old, decreasing amount of BMD speeds up for both females and males. Similar to previous studies some of the sex and race groups show significant difference in their BMD. Since heteroscedasticity is present among the age groups, different than the previous epidemiologic studies, we categorized the age groups by race and gender to investigate their BMD by the Tukey-Cramer confidence intervals. We used the 30–39-year age group for females and 20–29-year age group for males as the reference group because in all race groups bone mineral density reaches its peak level and then it starts to decrease gradually in these age groups. As we pointed out before our goal is to detect in which age groups bone mineral density starts to differ significantly from the reference group. Knowing this will help to detect early osteoporosis in different race groups.

Peak bone density (PBD) is probably the result of interaction between endogenous (heredity, endocrine) and exogenous (nutrition, physical) factors. In fact the fastest growing and development of skeleton is between early childhood and late adolescence [20]. About 60% of the bone growth takes place during the adolescence [21]. According to [22], the earliest age to reach peak bone density is between 17 and 18 years old and latest age is around 35 years old for the females.

Increasing of bone mass is different for different skeleton parts during puberty. Bone mass reached the peak level before 20 years old at the proximal femur; however for total skeleton it takes place after 6–10 years. Although our study shows that this time is little later than 20 years old for females, this result can be considered as being consistent with the previous studies. In a recent study it is found that estimated age of peak was around age of 20 for trabecular BMD. However this study was applied only on Chinese females and measurements were made by the high resolution peripheral quantitative computed tomography (HR-pQCT). Our study reveals that in Non-Hispanic Black females in the periods of 20–29 and 30–39 years BMD is significantly higher than other race groups. This result is consistent with the other cohort-based work. For example, in a recent study [23], BMD is compared in older women across five racial/ethnic groups in four countries. Findings show substantial racial differences in BMD even within African or Asian origin individuals and highlight the contributing role of body weight and estrogen use to the geographic and racial variation in BMD.

Similar to females it is found that males start to lose their BMD with aging. Although a 60-year-old White man has a 25% lifetime risk for an osteoporotic fracture, in general osteoporosis is underdiagnosed and undertreated problem for men [24]. In this cross-sectional study [25], bone mineral density (BMD) measurements were performed in 1762 ambulatory subjects (678 men and 1084 women) aged 55 years and over from the Rotterdam Study. This study revealed that the rate of age-related bone reduction in the femoral neck appears to be approximately two times higher in women than in men. However, the 1-year mortality rate in men after hip fracture is twice that in women [26]. Diagnostic evaluation and treatment of men at high risk for fracture remain low, despite the prevalence of this condition in men. According to our figures for men, bone mass reaches the peak level during 20–29-year period and it relatively keeps that level during 30–39-year period and BMD level starts decreasing after 40 years old. Results indicate that as it is the case in females, males should be cautious for possible fractures after 40 years old. In case of serious BMD decreasing, some actions should be taken such as modification in life style, doing more exercise and having medical treatment.

Our study also indicates that Non-Hispanic Black males have significantly higher BMD than Mexican-American and Non-Hispanic White in all age groups. Black males even have higher BMD in 40–49-year age group than peak levels of Mexican-American and White males which occur between 30 and 39 years. These findings are compatible with literature that low BMD is rare among the Non-Hispanic Blacks compared to others. In [27] BMD is compared in 1,209 Black, Hispanic, and White men. Black men exhibited higher BMD than Hispanic or White men. It has been detected that BMD decreases were greatest among Hispanic.

Recently one of the epidemiologic studies showed that 21.3% of the patients are detected as low BMD in general, while this ratio is 36% among the Non-Hispanic Whites and 38% among the Mexican-Americans. Studies indicate that Non-Hispanic Black children have higher trabecular bone density and have higher bone size in appendicular skeleton than their male counterparts [28]. This conclusion is based on having high level absorbance of renal calcium and having resistant bone tissue against parathormone among the Non-Hispanic Blacks [29].

Since residual bone mineral at the age of 60–90 years is the net result of multifactors, and since there are no safe, effective ways to rebuild the osteoporotic skeleton, prevention by maximizing bone mass during skeletal growth and development and minimizing postmenopausal bone losses emerges as the crucial strategy. Consequently, knowledge of appropriate timing of peak bone mass and BMD is essential if preventive measures are to be adequately implemented [30].

In this study we grouped the BMD of individuals by their age, race, and gender. This study indicates when each group reaches its peak BMD level. It is important to increase this level to prevent future low BMD cases. Also with the result of this study we will be able to take necessary actions when BMD shows start of significant drop compared to its peak level.

## 4. Conclusion

This report presents bone measurement data from whole body DXA scans for persons aged 8 and over who participated in the 1999–2006 NHANES. One of the limitations of the present study is that we are not able to estimate the specific prevalence of osteoporosis and low bone mass in Asian or Hispanic groups since the data we have only provide information for Non-Hispanic Whites, Non-Hispanic Blacks, and Mexican-Americans.

However interpretation of the Tukey-Cramer confidence intervals provides the following conclusions. For the Mexican-American females the peak bone density is for 30–39-year group. This group does not differ from the age groups of 20–29 years and 40–49 years. However BMD level of 30–39 years (X5) differs from the age groups of 16–19 years and 50–59 years. These results suggest that, for Mexican-American females, individual should continue to build strong bones until their early twenties and should start to be examined after 50 years old for sudden drops in their BMD.

For Non-Hispanic White females the peak time for bone density is the same as Mexican-American females, 30–39 years. However the age group of 30–39 years is different than that of 20–29 years with respect to their BMD. That means that this group should continue to build strong bones until their late twenties.

For the Non-Hispanic Black females the peak time of BMD is 30–39 years too. And this group is not statistically different than their 40–49 years. However BMD drops dramatically for the 50–59-year age group when it is compared with the age groups of 30–39 years and 40–49 years.

Different than the females, males reach the peak BMD during their 20–29 years. And this group is different than 16–19 years in all races. Mexican-Americans male and Non-Hispanic White males intend to keep their BMD level during their 30–39 years and 40–49 years the same as their female counterparts. However Non-Hispanic Black males show statistically significant difference in their 40–49-year age group. Although osteoporosis is not common among Non-Hispanic Black community, the sudden drop in 40–49-year age group of males should be examined in more detail in the future studies.

## Figures and Tables

**Table 1:** Total body mineral density (g/cm^2^) of Mexican-American females aged 8 years and over.

Female (Mexican-American)	Sample size	Mean	Standard deviation	Does Tukey-Kramer C. I. include zero?	
(X1) 8–11 years	471	0.820	0.091	(X5–X1)	×
(X2) 12–15 years	821	1.006	0.092	(X5–X2)	×
(X3) 16–19 years	692	1.074	0.089	(X5–X3)	×
(X4) 20–29 years	364	1.102	0.084	(X5-X4)	✓
(X5) 30–39 years	298	1.121	0.097	X5	✓
(X6) 40–49 years	372	1.121	0.092	(X5-X6)	✓
(X7) 50–59 years	194	1.059	0.125	(X5–X7)	×
(X8) 60–69 years	375	0.997	0.113	(X5–X8)	×
(X9) 70–79 years	154	0.937	0.105	(X5–X9)	×
(X10) 80 years and over	47	0.885	0.098	(X5–X10)	×

**Table 2 tab2:** Total body mineral density (g/cm^2^) of Non-Hispanic White females aged 8 years and over.

Female (Non-Hispanic White)	Sample size	Mean	Standard deviation	Does Tukey-Kramer C. I. include zero?	
(X1) 8–11 years	383	0.828	0.082	(X5–X1)	×
(X2) 12–15 years	576	1.003	0.092	(X5–X2)	×
(X3) 16–19 years	543	1.087	0.084	(X5–X3)	×
(X4) 20–29 years	583	1.109	0.079	(X5-X4)	×
(X5) 30–39 years	612	1.131	0.089	X5	✓
(X6) 40–49 years	668	1.127	0.096	(X5-X6)	✓
(X7) 50–59 years	639	1.091	0.099	(X5–X7)	×
(X8) 60–69 years	644	1.040	0.107	(X5–X8)	×
(X9) 70–79 years	429	0.977	0.104	(X5–X9)	×
(X10) 80 years and over	457	0.924	0.105	(X5–X10)	×

**Table 3 tab3:** Total body mineral density (g/cm^2^) of Non-Hispanic Black females aged 8 years and over.

Female (Non-Hispanic Black)	Sample size	Mean	Standard deviation	Does Tukey-Kramer C. I. include zero?	
(X1) 8–11 years	490	0.871	0.090	(X5–X1)	×
(X2) 12–15 years	737	1.071	0.097	(X5–X2)	×
(X3) 16–19 years	609	1.153	0.091	(X5–X3)	×
(X4) 20–29 years	297	1.186	0.092	(X5-X4)	✓
(X5) 30–39 years	333	1.196	0.095	X5	✓
(X6) 40–49 years	398	1.191	0.098	(X5-X6)	✓
(X7) 50–59 years	250	1.136	0.114	(X5–X7)	×
(X8) 60–69 years	306	1.096	0.113	(X5–X8)	×
(X9) 70–79 years	124	1.035	0.105	(X5–X9)	×
(X10) 80 years and over	58	0.979	0.129	(X5–X10)	×

**Table 4 tab4:** Total body mineral density (g/cm^2^) of Mexican-American males aged 8 years and over.

Male (Mexican-American)	Sample size	Mean	Standard deviation	Does Tukey-Kramer C. I. include zero?	
(X1) 8–11 years	468	0.833	0.075	(X4–X1)	×
(X2) 12–15 years	777	0.995	0.120	(X4–X2)	×
(X3) 16–19 years	783	1.142	0.103	(X4-X3)	×
(X4) 20–29 years	444	1.173	0.096	(X4)	✓
(X5) 30–39 years	337	1.162	0.094	(X4-X5)	✓
(X6) 40–49 years	381	1.155	0.101	(X4–X6)	✓
(X7) 50–59 years	174	1.142	0.109	(X4–X7)	×
(X8) 60–69 years	348	1.136	0.104	(X4–X8)	×
(X9) 70–79 years	164	1.106	0.107	(X4–X9)	×
(X10) 80 years and over	48	1.075	0.098	(X4–X10)	×

**Table 5 tab5:** Total body mineral density (g/cm^2^) of Non-Hispanic White males aged 8 years and over.

Male (Non-Hispanic White)	Sample size	Mean	Standard deviation	Does Tukey-Kramer C. I. include zero?	
(X1) 8–11 years	369	0.839	0.069	(X4–X1)	×
(X2) 12–15 years	578	0.995	0.111	(X4–X2)	×
(X3) 16–19 years	591	1.171	0.107	(X4-X3)	×
(X4) 20–29 years	617	1.210	0.099	(X4)	✓
(X5) 30–39 years	647	1.213	0.106	(X4-X5)	✓
(X6) 40–49 years	700	1.206	0.102	(X4–X6)	✓
(X7) 50–59 years	683	1.181	0.110	(X4–X7)	×
(X8) 60–69 years	623	1.167	0.114	(X4–X8)	×
(X9) 70–79 years	496	1.135	0.114	(X4–X9)	×
(X10) 80 years and over	398	1.107	0.115	(X4–X10)	×

**Table 6:** Total body mineral density (g/cm^2^) of Non-Hispanic Black males aged 8 years and over.

Male (Non-Hispanic Black)	Sample size	Mean	Standard deviation	Does Tukey-Kramer C. I. include zero?	
(X1) 8–11 years	469	0.884	0.077	(X4–X1)	×
(X2) 12–15 years	745	1.044	0.118	(X4–X2)	×
(X3) 16–19 years	757	1.233	0.116	(X4-X3)	×
(X4) 20–29 years	325	1.304	0.120	(X4)	✓
(X5) 30–39 years	310	1.292	0.114	(X4-X5)	✓
(X6) 40–49 years	366	1.258	0.131	(X4–X6)	×
(X7) 50–59 years	240	1.256	0.122	(X4–X7)	×
(X8) 60–69 years	303	1.240	0.115	(X4–X8)	×
(X9) 70–79 years	114	1.167	0.119	(X4–X9)	×
(X10) 80 years and over	36	1.178	0.156	(X4–X10)	×
